# Ebselen prevents cigarette smoke-induced gastrointestinal dysfunction in mice

**DOI:** 10.1042/CS20200886

**Published:** 2020-11-16

**Authors:** Gayathri K. Balasuriya, Mitra Mohsenipour, Kurt Brassington, Aleksandar Dobric, Simone N. De Luca, Kevin Mou, Huei Jiunn Seow, Chalystha Yie Qin Lee, Madushani Herath, Stanley M.H. Chan, Ross Vlahos, Elisa L. Hill-Yardin

**Affiliations:** 1 School of Health and Biomedical Sciences, RMIT University, Bundoora, Victoria 3083, Australia; 2 Department of Physiology, University of Melbourne, Parkville 3010, Australia

**Keywords:** cigarette smoking, COPD, ebselen, gastrointestinal, mice

## Abstract

Gastrointestinal (GI) dysfunction is a common comorbidity of chronic obstructive pulmonary disease (COPD) for which a major cause is cigarette smoking (CS). The underlying mechanisms and precise effects of CS on gut contractility, however, are not fully characterised. Therefore, the aim of the present study was to investigate whether CS impacts GI function and structure in a mouse model of CS-induced COPD. We also aimed to investigate GI function in the presence of ebselen, an antioxidant that has shown beneficial effects on lung inflammation resulting from CS exposure. Mice were exposed to CS for 2 or 6 months. GI structure was analysed by histology and immunofluorescence. After 2 months of CS exposure, *ex vivo* gut motility was analysed using video-imaging techniques to examine changes in colonic migrating motor complexes (CMMCs). CS decreased colon length in mice. Mice exposed to CS for 2 months had a higher frequency of CMMCs and a reduced resting colonic diameter but no change in enteric neuron numbers. Ten days cessation after 2 months CS reversed CMMC frequency changes but not the reduced colonic diameter phenotype. Ebselen treatment reversed the CS-induced reduction in colonic diameter. After 6 months CS, the number of myenteric nitric-oxide producing neurons was significantly reduced. This is the first evidence of colonic dysmotility in a mouse model of CS-induced COPD. Dysmotility after 2 months CS is not due to altered neuron numbers; however, prolonged CS-exposure significantly reduced enteric neuron numbers in mice. Further research is needed to assess potential therapeutic applications of ebselen in GI dysfunction in COPD.

## Introduction

Cigarette smoking (CS) is a major cause of mortality and disease, including chronic obstructive pulmonary disease (COPD). It has been shown that chronic smokers have gut dysfunction [1]. COPD is characterised by progressive airflow obstruction and enhanced inflammation and is the fourth leading cause of death worldwide [2]. CS is a major cause of COPD and extrapulmonary effects including those involving the gastrointestinal (GI) tract on patient health have also been demonstrated [3,4]. The GI tract is recognized as a major site of extrapulmonary dysfunction that impacts COPD patients and quality of life [3].

CS exposure has differing effects on GI disorders such as Crohn’s disease and ulcerative colitis (UC). CS exposure and COPD are risk factors for Crohn’s disease that is characterised by chronic mucosal inflammation and compromised intestinal barrier function [5]. In contrast, chronic active smoking has been shown to decrease the severity of UC and the disease progression is less advanced in smokers than non-smokers [6,7]. Ex-smokers have an increased risk of developing UC and severe symptoms needing surgery [6]. However results from another population-based cohort study has revealed COPD as a risk factor for both UC and Crohn’s disease, suggesting that there is a significant interaction between both COPD and the structure and function of the GI tract [5]. Interestingly, CS exposure dose-dependently increased adenoma formation in mice with inflamed mucosa induced by 3% dextran sulphate sodium (DSS) [8].

Recently, it has been shown that CS exposure alters GI structure and function in mice. Research using a CS-induced COPD model in C57BL/6 mice showed a phenotype of Crohn’s disease that includes increased gut mucosal tissue hypoxia and microvasculature inflammation, epithelial cell turnover and muscle layer thickness [9]. This group also reported increased intestinal permeability in the colon of these mice [9]. Interestingly, when exposed to an inflammatory insult (2,4,6-trinitrobenzenesulfonic acid; TNBS) to induce colitis, these mice showed exacerbated intestinal pathology suggesting CS is an important risk factor for GI dysfunction.

Mucus production is critically involved in regulating GI barrier function and inflammatory responses. However, few studies have assessed for alterations in GI mucus production in response to CS exposure, or nicotine specifically. In non-smokers, transdermal application of nicotine had no effect on mucin (the major component of GI tract mucus) gene expression in UC patients [10]. Additionally, in a preclinical randomised controlled study using New Zealand white rabbits, increased mucus production was seen in the rectal mucosa following subcutaneous administration of high doses (2 mg/Kg/day for 14 days) of nicotine [11]. However, it is unclear whether mucus properties within the GI tract are altered in the BALB/c mouse model of CS-induced COPD.

CS alters the immune system including immune responses within the GI tract. In a human trial analysing gut mucosal biopsies of male participants aged 20–65 including 21 chronic smokers and 21 non-smoker controls, lower levels of IgA (the major players in defense against bacteria and viruses adhering to the GI mucus), but not IgG and IgM were observed, likely indicating an alteration of immune responses (Srivastava et al., 1991). Alterations in the density of immunoregulatory T cells (which are important in cell-mediated immunity and the activation of the immune defense system against pathogens and infections) in the peripheral circulation were observed in a subpopulation of COPD patients with altered pulmonary function [12].

The nicotine component of cigarette smoke could elicit a proinflammatory response via the induction of oxidative stress. In mouse colonic mucosa, subcutaneous nicotine reduces levels of the proinflammatory cytokines interleukin-1 β (IL-1β) and tumor necrosis factor-α (TNFα) [13]. Muscularis macrophages reside in close proximity to myenteric neurons and are responsible for the phagocytosis of dead neurons to maintain neuronal homeostasis [14,15]. These macrophages also make close connections with myenteric neurons that regulate intestinal motility. Furthemore, in aged mice, these macrophages can shift their function towards a proinflammatory phenotype to cause inflammation-mediated degeneration of the enteric nervous system (ENS) [16].

Within the GI tract, the ENS contains a complex network of neuronal subpopulations including nitric oxide synthase (NOS)-expressing cells that release the major enteric inhibitory neurotransmitter, nitric oxide (NO). The ENS also contains a high density of nicotinic receptors, one of the main sites of action of the major excitatory neurotransmitter Acetylcholine (ACh) that regulates GI tract secretion and motility. Since CS influences intestinal inflammation, and changes in myenteric NOS neuronal proportions have been reported in both patients and animal models of intestinal inflammation [17–19], we examined for changes in myenteric NOS neuron numbers on exposure to chronic CS-exposure. It is well established that CS impacts GI secretion [20] and permeability [9]; however the effects of CS on gut motility, which is predominantly regulated by myenteric neurons, are largely unknown.

We have previously shown that the antioxidant ebselen inhibits CS-induced lung inflammation [21], Influenza A virus (IAV)-induced lung inflammation [22] and IAV-mediated exacerbation of CS-induced lung inflammation [23] in mice. Moreover, we have shown that the increases in bronchoalveolar lavage fluid (BALF) macrophages caused by CS alone or in the presence of IAV were significantly reduced by ebselen treatment. We therefore aimed to investigate where ebselen has positive effects on GI function in the CS-induced mouse model of COPD.

Given that CS impairs gastrointestinal function, it is necessary to investigate changes in the enteric nervous system that potentially contribute to dysmotility. Here, we examined for changes in gut anatomy and physiology including characterisation of muscularis macrophages, enteric neuronal subpopulations and colonic motility in the BALB/c mouse model of CS-induced COPD to understand the relationship between CS, COPD and gut dysfunction. Moreover, we assessed the effects of ebselen on gut motility in control and CS-exposed mice.

## Methods

### Mice

All experiments were completed to meet the Australian Code of Practice for the Care of Experimental Animals and the ARRIVE Guidelines, following RMIT University Animal Ethics Committee approval (Animal Ethics Application Number 1521 & 1533). Seven-week-old male BALB/c mice were obtained from the Animal Resource Centre Pty Ltd (Perth, WA, Australia). All mice were housed under standard laboratory housing conditions, with a 12-h light cycle (7 am to 7 pm), an ambient temperature of 21°C, with humidity between 40 and 60% in sterile micro-isolator cages (Able Scientific, Australia) with *ad libitum* access to both water and standard mouse chow (Glen Forest Speciality Foods, Australia). Mice were housed at the RMIT Animal Facility and experiments were conducted at the School of Health and Biomedical Sciences, RMIT University, Bundoora, Victoria 3083, Australia.

### Cigarette smoke exposure and drug/placebo administration

Mice were placed into an 18L Perspex chamber (The Plastic Man, Huntingdale, VIC, Australia) in a standard biosafety cabinet (Aircare Extraction Systems Ltd, Clayton, VIC, Australia). Mice were exposed to either room air (sham) or mainstream CS generated from 9 Winfield Red Cigarettes (16 mg or less of tar, 15 mg or less of CO and 1.2 mg or less of nicotine, Philip Morris, Moorabbin, VIC, Australia) for 5 days a week for either 2 (*n* = 28/group) or 6 months (*n* = 20/group), with a sub-group from the 2 month study subject to a 10-day CS cessation (*n* =14). CS was delivered over three sessions of three cigarettes per day, over 1 h time periods at 9 am, 12 noon and 3 pm. CS generation occurs in 60 ml tidal volumes over a 10 s timed draw, mimicking normal smoke inhalation and burn rate [24]. Sham-exposed control mice were placed into an identical Perspex chamber, but did not receive CS. Separate cohorts of mice were treated with 10 mg/kg of ebselen (Sapphire Bioscience, Australia) prepared in 5% w/v carboxymethyl (CM)-cellulose (Sigma-Aldrich, U.S.A.) or vehicle (5% CM-cellulose; CMC) alone via oral gavage daily 1 h prior to initial CS. This method of CS-exposure has been used extensively as it recapitulates the hallmark clinical characteristics of human COPD, including pulmonary inflammation and oxidative stress, as well as the ability to impair lung function and promote comorbid pathologies such as skeletal muscle wasting and stroke [25–27]. After 2 and 6 months of CS exposure, mice were euthanised with > 150 mg/kg intraperitoneal (i.p.) sodium pentobarbital and additional specific experiments are described below.

### Gut anatomy and histopathology

The entire gut was dissected from the mice and the length of small intestine and colon was measured, and caecum weight recorded.

Following dissection, 1 cm length of the proximal colon and 1 cm length of the proximal most jejunum was fixed in 4% formaldehyde and cryoprotected in 30% sucrose before processing to obtain frozen cross-sections of 10 μm thickness. Sections were then stained with hematoxylin and eosin (H&E) to identify tissue structural features. After imaging (using Olympus Australia Pty. Ltd.; Melbourne, Australia), length of villi, width of villi and crypt depths were measured using ImageJ software (ImageJ 1.52a, NIH).

Mid colon samples containing a faecal pellet were selected and fixed in methanol-Carnoy’s fixative (composition, %: absolute methanol 60, chloroform 30, glacial acetic acid 10) and cryoprotected in 30% sucrose before obtaining 10 μm thick frozen transverse sections for 6 months smoked tissues. After fixation for 2 months, CS mid colons were processed to obtain paraffin sections. Sections were then stained with Alcian Blue pH 2.5 (Sigma-Aldrich, Germany) followed by counterstaining with Nuclear Fast Red (Sigma-Aldrich, Germany). Slides were imaged using Olympus slides scanner microscope (Olympus Australia Pty. Ltd.; Melbourne, Australia). The thickness of the mucus layer was measured. Specifically, this was done by first selecting a region of interest and tracing the stained mucus layer using FIJI ImageJ (ImageJ 1.52a, NIH) [28]. The area of the identified mucus layer was then normalised by dividing the area measurement against the length of the epithelial perimeter of the selected region of interest. Therefore, the area of the mucus thickness/unit length of the perimeter was calculated as the normalised mucus layer thickness. A similar procedure was followed to measure the area of the smooth muscle layer/unit length of the perimeter from the same images.

### Video imaging of gut motility

Colonic motility was analysed in mice from the sham exposed, 2 months smoked, 10 days cessation after 2 months smoking, as well as CS and sham mice that received Ebselen and vehicle treatment for 2 months. The entire colon was excised immediately upon sacrifice and placed into an organ bath containing warmed (36°C) KREBS physiological saline solution (composition, mM: NaCl 118, KCl 4.6, NaH_2_PO_4_ 1, NaHCO_3_ 25, MgSO_4_ 1.2, D-glucose 11, CaCl_2_ 2.5; bubbled with 95% O_2_: 5%CO_2_). Physiological saline was continuously superfused through the organ bath at a flow rate of approximately 6 ml min^−1^ [29,30]. The oral end of the tissue was connected to a reservoir of physiological saline, the anal end to an outflow tube that provided a back pressure of 3-4 cm H_2_O. Video imaging analysis of colonic motility was conducted as previously described [30,31]. Briefly, colonic motility was recorded *in vitro* using a Logitech camera (QuickCam Pro 4000; I-Tech, Ultimo, NSW, Australia) mounted directly above the organ bath. In-house software (Scribble 2.0) and a purpose-built Matlab (2016b) plugin, Analyse 2.0, were used to convert recorded video segments (15 min duration) to spatiotemporal maps where the diameter of the colon is mapped (as a heat map) along the length of the segment as a function of time. For spatiotemporal maps, the *x* axis represents increasing time, and the length of colonic segment is represented along the *y* axis. The diameter along the colon is pseudocoloured, such that blue-green pixels indicate relaxed tissue and yellow-red pixels identify constricted regions (Figure 4A).

Two indices of neurally mediated colonic motor activity were analysed: colonic migrating motor complexes (CMMCs) defined as spontaneous constractions originating at the oral end of the colon that propagate more than half of the length of the tissue, and the resting colonic diameter (diameter of the colon between CMMCs when the colon is quiescent).

CMMC frequency, the speed of CMMC propagation and resting gut diameter were measured using Analyse2 software as previously reported (Swaminathan et al., 2015; Gwynne et al., 2004). CMMC frequency was manually counted from spatiotemporal maps. We measured resting colonic diameter at timepoints between contractions in the presence of constant luminal pressure. Hence, we recorded the mean gut diameter at these timepoints at a location equating to 66% of the full colonic length from the oral end of the preparation. The experimental protocol for motility studies consisted of a 30 min equilibration period followed by a 1 h recording (consisting of four 15-min duration videos) of baseline colonic motility.

### Immunofluorescence

Immunofluorescent staining for the pan neuronal marker Hu (1:10000, a gift from Dr Lennon, U.S.A.), the neuronal Nitric Oxide Synthase (1:400, #AB1529, Millipore, U.S.A.) and pan macrophage marker Iba1 (1:500, #ab178847, Abcam, U.S.A.) were conducted in colon myenteric plexus preparations from 2 to 6 months smoked and sham mice. Wholemount preparations of proximal colon preparations and jejunum were prepared and immunohistochemistry conducted as reported previously (Leembruggen and Balasuriya et al., 2019). Briefly, tissue preparations were incubated in primary antisera overnight at 4°C, washed three times in phosphate-buffered saline (PBS; pH7.2) and incubated in corresponding fluorescent-tagged secondary antisera for 2 hrs (Donkey anti sheep Alexa 488, #A11015 Molecular probes used at 1:400; Donkey anti human Alexa 594, #A11001 Molecular probes used at 1:5000; Donkey anti rabbit Alexa 647, #132485 Jackson Immuno-Research laboratories used at 1:400). Tissue preparations were subsequently washed in PBS and mounted on glass slides for analysis. Images were captured on a confocal electron microscope (Nikon Confocal Microscope: A1; Version 4.10). Digital images for Hu and NOS labelling were quantitatively analysed using ImageJ software (NIH Bethesda etc U.S.A.). IMARIS software (Imaris x64 9.1.0; Bitplane AG, U.K.) was used to undertake 3D reconstruction of individual cells and for the quantification of density, volume and sphericity of Iba1 immunoreactive cells in myenteric plexus preparations.

### Statistical analysis

Statistical analysis was performed using GraphPad Prism (Version 8.3.1, CA). CMMC numbers were analyzed using a Mann–Whitney test. Two‐tailed, unpaired *t* tests and one‐way ANOVA was used for the rest of the data analysis. Data are presented as mean ± SEM.

## Results

### Both 2 and 6 months CS exposure results in irreversible anatomical changes in the GI tract of mice

**Figure 1:**
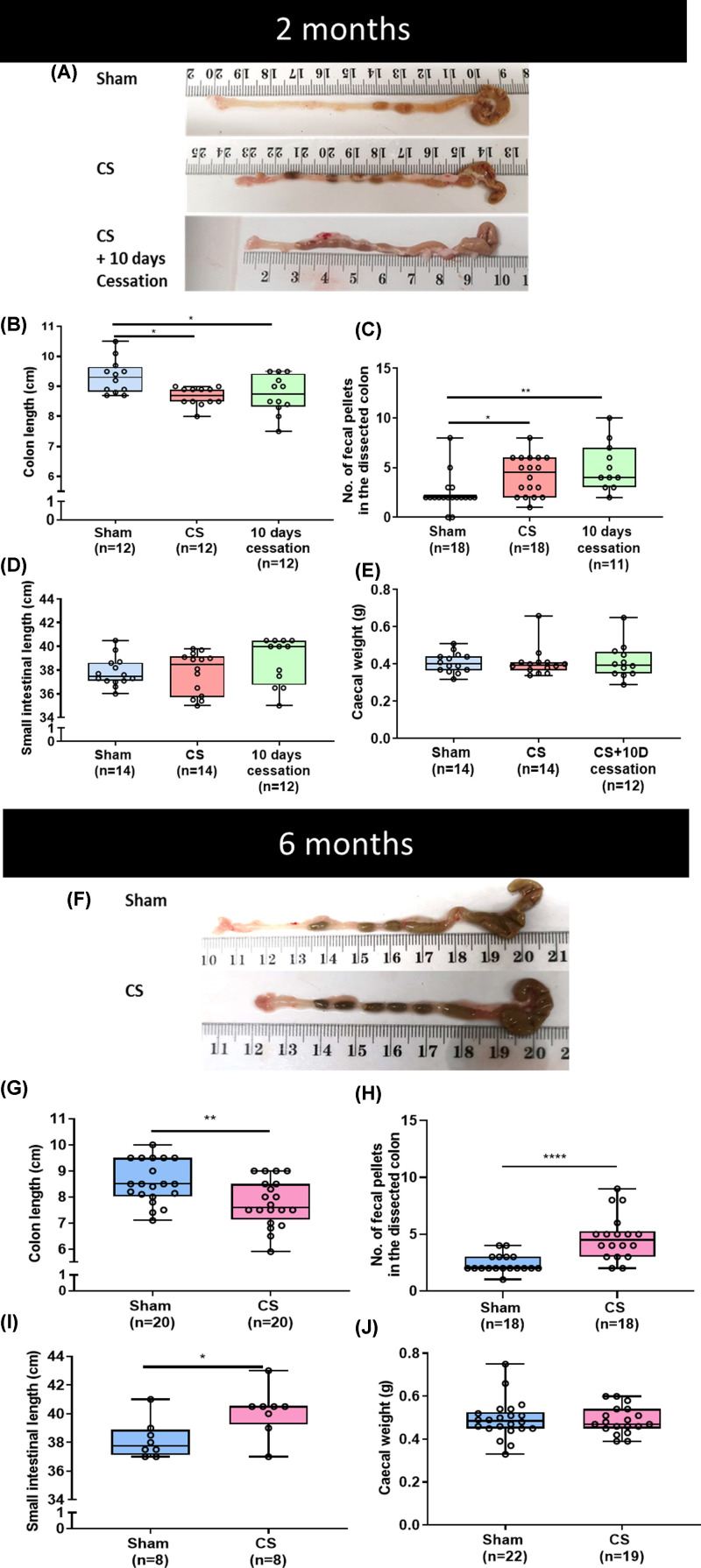
Anatomical changes in the GI tract after chronic CS exposure Representative images of mouse colon at 2 and 6 months CS exposure (**A** and **F**, respectively). Shortening of the colon was observed after 2 months (**B**) and 6 months (**G**) CS exposure. Number of faecal pellets increased within the dissected colon at 2 months (**C**) and 6 months (**H**) CS mice. SI length was unaffected after 2 months (**D**) and 6 months CS (**I**) increased the SI length. Caecal weights were not changed at 2 months (**E**) and 6 months (**J**) of CS exposure. CS cessation for 10 days following 2 months CS did not rescue altered colon length and faecal pellet counts (B and C); *P<0.05, **P<0.01, ****P<0.0001.

Mice exposed to CS for 2 and 6 months were compared with sham (room air exposed) mice at the respective time points. Furthermore, sham and CS mice at 2 months were compared with mice with 10 days of smoke cessation following 2 months CS exposure. A decrease in colon length was observed both after 2 and 6 months of CS exposure (Figure 1B: 2 months sham 9.32 ± 0.17 cm vs CS 8.67 ± 0.09 cm, *P*=0.012; Figure 1G: 6 months sham 9.25 ± 0.19 vs CS 8.62 ± 0.16, *P*=0.024). The decreased length persisted even after a 10-day CS cessation period following 2 months CS (Figure 1B: 8.74 ± 0.19, *P*=0.029 vs 2 months sham) but did not lead to further changes in colon length compared with the 2 months CS group (Figure 1B: *P*=0.936). The small intestinal length was unchanged after 2 months of CS; however, 6 months of CS exposure led to an increase in length (Figure 1D: 2 months sham 37.81 ± 0.47 cm vs CS 37.77 ± 0.33 cm, *P*=0.998; Figure 1I: 6 months sham 38.19 ± 0.47 cm vs CS 40.13 ± 0.59 cm, *P*=0.023). An increase in the number of faecal pellets within the dissected colon has previously been reported in animal models of intestinal dysmotility and inflammation [32,33] and we report a similar finding both after 2 and 6 months of CS exposure (Figure 1C: 2 months sham 2 ± 0.4 vs CS 4 ± 0.5, *P*=0.044; Figure 1H: 6 months sham 2 ± 0.2 vs CS 5 ± 0.5, *P*=0.0001). The increase was persistent even after ceasing smoking for 10 days following 2 months of CS exposure (Figure 1C: 5 ± 0.7, *P*=0.003 vs 2 months sham and similar to 2 months CS exposure *P*=0.699). Since the length of the colon could potentially be affected by the volume of faecal content due to widening of the colon in regions containing faecal pellets, we analysed the ratio of colon length to the number of faecal pellets. We found that the ratio of the colon length to the number of faecal pellets was reduced after CS exposure at both the 2- and 6-month timepoints (2 months sham 4.05 ± 0.39 vs CS 2.79 ± 0.36, *P*=0.033 and 6 months sham 3.37 ± 0.38 vs CS 1.84 ± 0.21, *P*=0.002; Supplementary Figure S3). Altered caecal weight was previously reported in mouse models of inflammation [34,35]; however no difference in caecal weight was observed following either 2 or 6 months of CS exposure (Figure 1E: 2 months sham 0.41 ± 0.041 vs CS 0.21 ± 0.02 g, *P*=0.997 and Figure J: 6 months sham 0.46 ± 0.045 vs CS 0.45 ± 0.011, *P*=0.857).

### Typical histopathology alongside a reduced mucus layer thickness in the mid colon was observed following both 2 and 6 months of CS exposure

**Figure 2:**
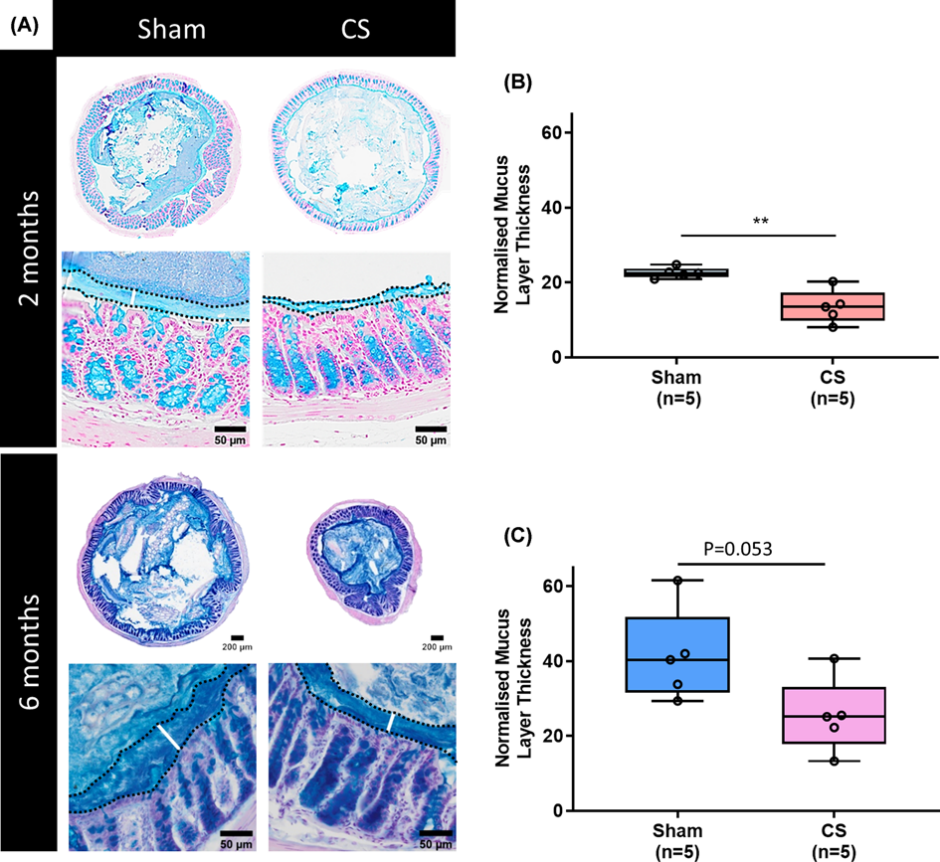
Reduced mucus thickness in the mid colon of CS mice (**A**) Images are of faecal pellet-containing mid colon preparations of sham and CS mice stained with Alcian Blue to measure the thickness of the mucus layer. (**B**) Two months of CS led to a reduction in mucus layer thickness, **P<0.001. (**C**) A near significant reduction (*P*=0.053) was observed in the mucus layer thickness after 6 months of CS.

Following histological analysis, crypt depth in the jejunum and colon was unchanged after 2 and 6 months of CS exposure. However, a statistically nonsignificant shortening of crypt depth was observed after CS exposure (2 months sham 171.4 ± 4.63 μm vs CS 162.0 ± 7.35 μm, *P*=0.311; 6 months sham 101.5 ± 9.31 μm vs CS 99.26 ± 3.45 μm, *P*=0.825; Supplementary Figure S1). Mucus layer thickness was reduced after 2 months of CS exposure (Figure 2B: sham 22.61 ± 0.63 μm vs CS 13.54 ± 1.99 μm, *P*=0.0025). There was a trend for a reduction in mucus thickness after 6 months of CS exposure (Figure 2C: sham 41.42 ± 5.15 μm vs CS 25.40 ± 4.141 μm, *P*=0.053). Normalised smooth muscle layer thickness of these samples was unaffected in mice exposed to CS during 2 and 6 months (2 months sham 64.25 ± 10.3 μm vs CS 42.61 ± 7.05 μm, *P*=0.122 and 6 months sham 63.27 ± 3.03 μm vs CS 47.91 ± 12.84 μm, *P*=0.235; Supplementary Figure S2).

### Prolonged cigarette smoke exposure leads to significant alterations in myenteric neurons

**Figure 3:**
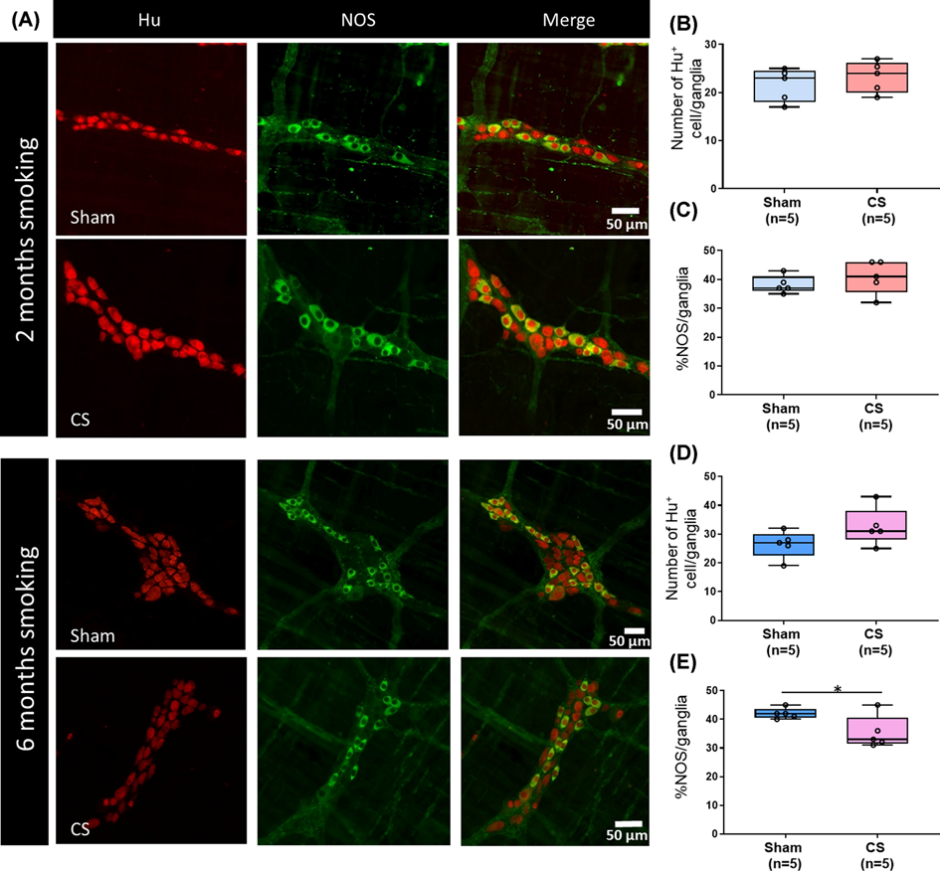
Prolonged exposure to CS leads to changes in ENS composition (**A**) Representative images are of whole mount preparation of mouse distal colon myenteric plexus preparations, immuno-stained with pan neuronal marker Hu (in red) and nitric oxide synthase (nNOS; in green). Two months CS had no effect on total neuron numbers or %NOS neurons per ganglion (**B** and **C**). Six months CS led to a decrease in the % of NOS cells per ganglion (**E**; *P<0.05) but had no effect on total number of neurons per ganglion (**D**).

**Figure 4:**
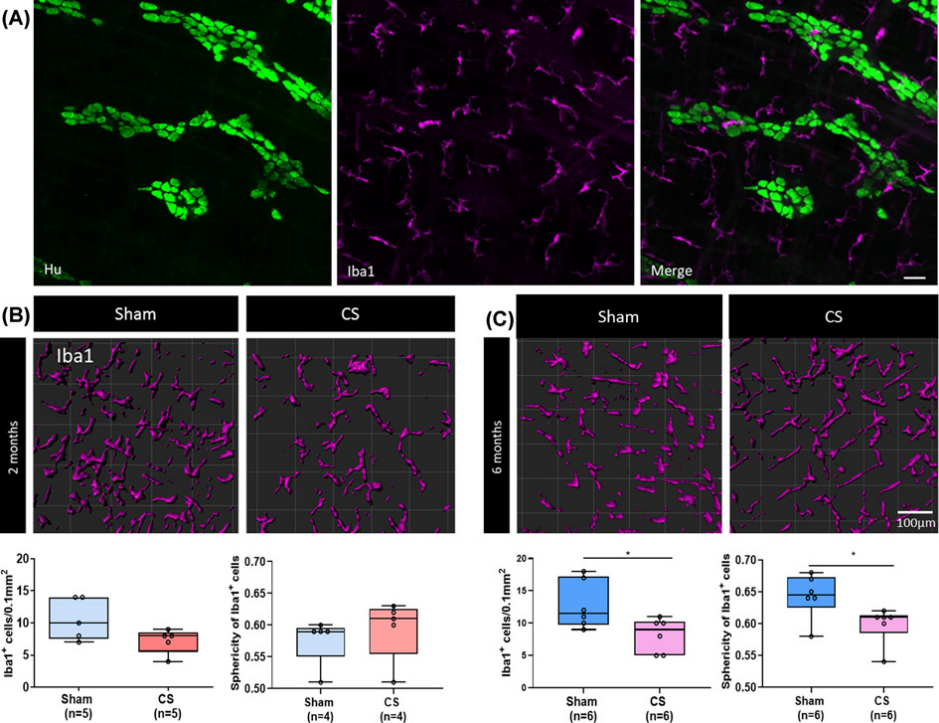
Long-term CS exposure alters muscularis macrophage density and morphology in mouse myenteric plexus preparations (**A**) Representative image illustrating the expression of muscularis macrophages labelled with Iba1 (in magenta) with respect to myenteric neurons labelled with pan neuronal marker Hu (in green), in a wholemount myenteric plexus preparation of a mouse distal colon (scale bar = 50 μm). (**B**) No significant changes in Iba1^+^ cell density and sphericity of muscularis macrophages after 2 months CS exposure. (**C**) Six months of CS led to a decrease in Iba1^+^ macrophage density and sphericity. *P<0.05.

Six months CS exposure decreased the percentage of NOS-expressing neurons per ganglion (Figure 3E: sham 42 ± 1% vs CS 35 ± 3%, *P*=0.039) whereas 2 months CS exposure had no significant effect (Figure 3C: sham 38 ± 1% vs CS 41 ± 3%, *P*=0.401). However, both 2 and 6 months CS exposure had no effect on the total number of neurons per myenteric ganglion (Figure 3B: 2 months sham 22 ± 2 vs 2 months CS 23 ± 2, *P*=0.45; Figure 3D: 6 months sham 24 ± 2 vs 6 months CS 33 ± 3, *P*=0.124). Iba1, which is a pan macrophage marker, is expressed in close proximity to myenteric neurons as well as between ganglia (Figure 4A). Iba1+ expressing muscularis macrophage density was reduced after 2 months of CS exposure; however, this was not statistically significant (Figure 4B: sham 11 ± 2 vs CS 7 ± 2 Iba1+ cells/0.1 mm^2^, *P*=0.191). The density of muscularis macrophages was reduced after 6 months of CS exposure (Figure 4C: sham 13 ± 2 vs 8 ± 1 Iba1+ cells/0.1 mm^2^ area, *P*=0.047). In mice exposed to 6 months of CS, the sphericity of muscularis macrophages was also reduced (Figure 4C: sham 0.64 ± 0.01 vs CS 59 ± 0.01, *P*=0.036). In contrast, the morphology of Iba1-expressing macrophages was unchanged after 2 months of CS exposure when compared with the respective sham group (Figure 4B: sham 0.58 ± 0.02 vs CS 0.59 ± 0.02, *P*=0.527).

### Two months of cigarette smoke exposure leads to increased colonic motility

**Figure 5:**
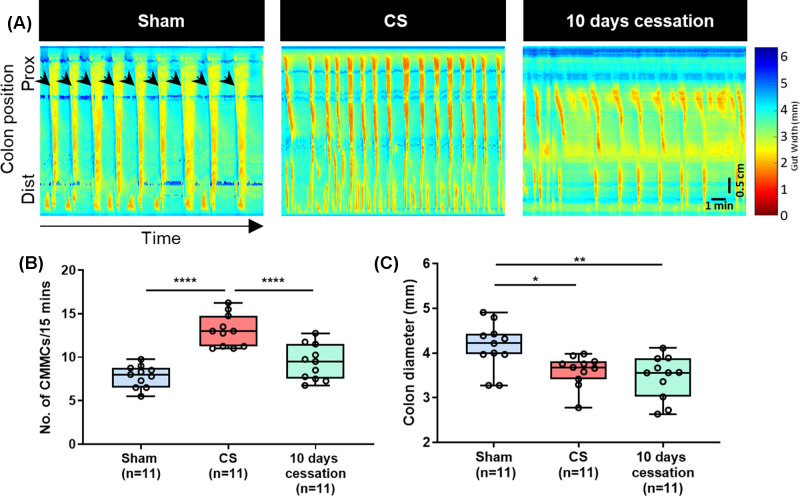
CS exposure for 2 months alters colonic contractions (**A**) Spatiotemporal maps representing colonic motility in sham, 2 months CS and 2 months CS mice with 10 days cessation. *X* axis represents time. *Y* axis represents the gut position from proximal colon to distal colon. Arrows indicate individual colonic migrating motor complexes (CMMCs). Horizontal scale bar = Time (1 min). Vertical scale bar = 0.5 cm (length of colon). The colour scale indicates the width of the colon for each captured frame during the 15 min recording. (**B**) CMMC frequency was increased after 2 months of CS compared to sham mice (*P*<0.0001) and 10 days of cessation after two months of CS reversed this effect (*P*>0.05 vs sham and *P*<0.0001 vs CS). (**C**) Two months CS reduced the resting colon diameter (*P*<0.05) and cessation for 10 days did not reverse the reduction in colon diameter (*P*<0.01 vs sham). *P<0.05, **P<0.01, ****P<0.0001.

Neurally regulated colonic motor complexes were significantly increased following 2 months of CS exposure (Figure 5A,B: sham 8 ± 0.4 CMMCs/15 min vs CS 13 ± 0.6 CMMCs/15 min, *P*<0.0001). Furthermore, the speed of these contractions was increased (sham 2.33 ± 0.16 mm/s vs CS 3.24 ± 0.21 mm/s, *P*=0.0052; data not shown). Resting colonic diameter, an indicator of colonic muscle tone, was reduced in mice that were exposed to CS for 2 months (Figure 5C: sham 4.14 ± 0.2 mm vs CS 3.60 ± 0.1 mm, *P*=0.018). 10 days of CS cessation post 2 months of CS exposure reversed the effects of CS with respect to contraction frequency (Figure 5B: 10 days cessation 9 ± 0.60 CMMCs/15 min, *p* = 0.208 vs sham and different from CS mice *P*=0.0016). However, 10 days of cessation did not reverse the reduced colon diameter (Figure 5C: 10 days cessation 3.45 ± 0.07 mm, *P*=0.0015 vs sham and *P*=0.815 vs CS).

### Treatment with ebselen improves CS-induced reduction colonic diameter

**Figure 6:**
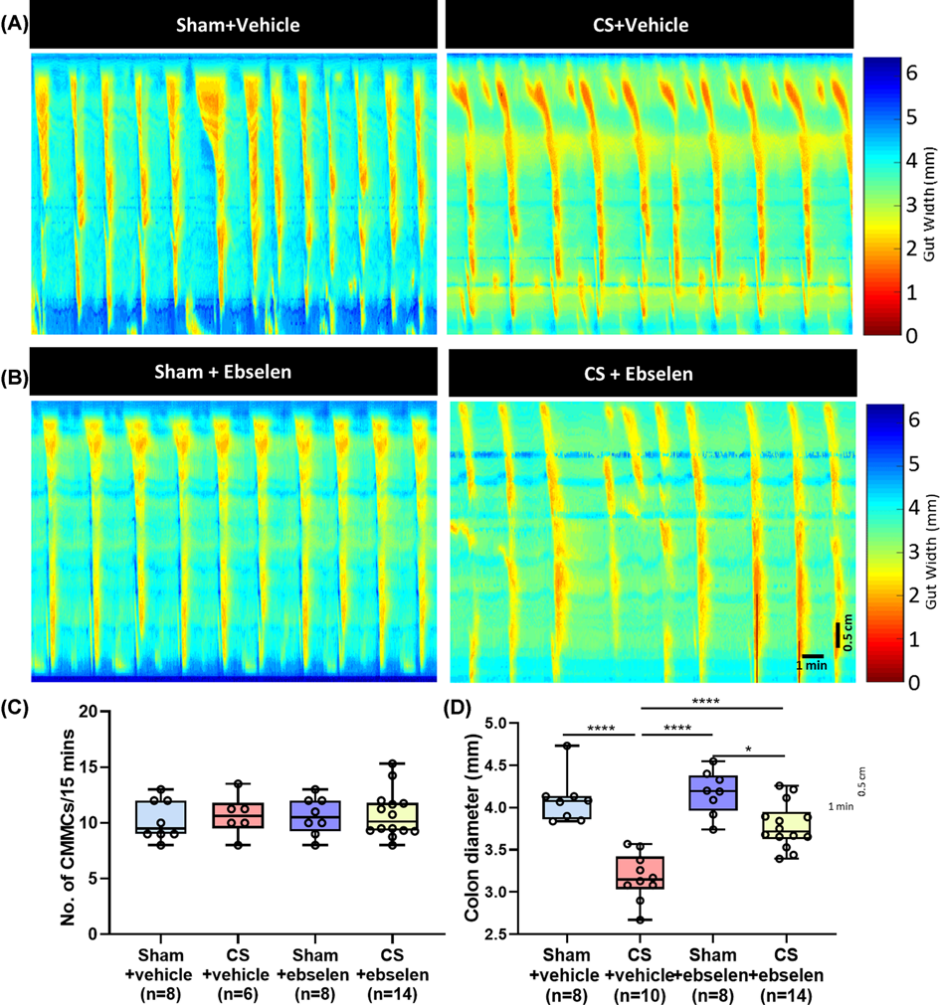
Treatment with ebselen has positive effects on colonic diameter changes induced by CS (**A**) Representative spatiotemporal maps of sham and 2 months CS mice treated with vehicle (5% CM-cellulose in PBS). (**B**) Representative spatiotemporal maps of sham and 2 months CS mice treated with ebselen (10 mg/kg). (**C**) Both vehicle and ebselen treatment restored CMMC frequency of CS exposed mice to sham levels (*P*>0.05). (**D**) Vehicle-treated CS mice had a reduced colonic diameter compared with sham mice (****P<0.0001) and ebselen-treated CS mice had an increased colonic diameter compared with CS+vehicle mice (****P<0.0001); horizontal scale bar = Time (1 min); vertical scale bar = 0.5 cm (length of colon). *P<0.05.

Ebselen treatment restored contraction frequency changes in the colon caused by CS (Figure 6A,B; Frequency: sham+ebselen 11 ± 0.6 CMMCs/15 min vs CS+ebselen 11 ± 0.6 CMMCs/15 min, *P*=0.877). However, the CS+vehicle group also reversed the colonic contraction frequency (Figure 6C: 11 ± 0.7 CMMCs/15 min, *P*>0.05 compared with all other groups). The colonic diameter was smaller in CS mice treated with ebselen compared with sham mice treated with ebselen (Figure 6D: sham+ebselen 4.18 ± 0.09 mm vs CS+ebselen 3.79 ± 0.07 mm, *P*=0.003). However, the average colon diameter in mice from the CS+ebselen group was greater than for mice in the 2 months CS group treated with vehicle (Figure 6D: CS+ebselen 3.79 ± 0.072 mm vs CS+vehicle 3. 18 ± 0.081 mm, *P*<0.0001). These findings suggest a positive effect of ebselen on the reduced colonic diameter induced by CS.

### Ebselen or vehicle did not reverse altered GI anatomical parameters induced by CS

**Figure 7:**
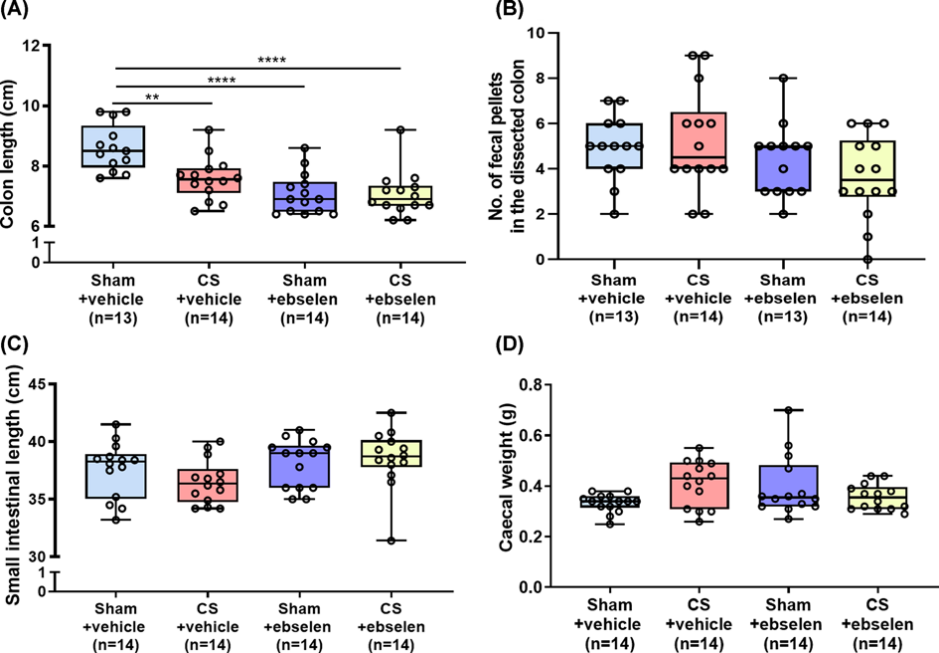
Treatment with ebselen did not reverse altered GI anatomical parameters Effect of ebselen and vehicle treatment on colon length (**A**), number of faecal pellets (**B**), small intestinal length (**C**) and caecal weight (**D**). Treatment with vehicle or ebselen did not reverse reduced colonic length caused by CS, but ebselen treatment reduced colon length in the sham group (**A**). Both Ebselen and vehicle treatment did not rescue increased faecal pellet numbers due to CS (**B**). Caecal weight was unaffected due to vehicle or ebselen treatment (**D**; **P<0.01, ****P<0.0001).

Treatment with vehicle (CMC) or ebselen did not reverse reduced colonic length following 2 months CS (Figures 1B and 7A: Sham+vehicle 8.61 ± 0.21cm vs CS+vehicle 7.59 ± 0.19 cm, *P*=0.003; vs CS+ebselen 7.06 ± 0.20 cm, *P*<0.0001). Treatment with the CMC vehicle reduced the colon length of sham mice when compared to sham control mice (sham controls 9.33 ± 0.17 cm (Figure 1B) vs sham+CMC 8.561 ± 0.21 cm (Figure 7A), *P*=0.0155). CS exacerbates CMC-induced colon shortening (Sham+CMC colon lengths: 8.561 ± 0.21 cm vs CS+CMC colon lengths: 7.59 ± 0.19 cm; *P*=0.003; Figure 7A). However, ebselen treatment did not further reduce colon length when compared to the CS+CMC treated group (CS+CMC colon length: 7.59 ± 0.19 cm vs CS+ebselen colon length: 7.06 ± 0.20 cm, *P*=0.244). Ebselen treatment led to a reduction in colon length in sham-treated mice (Figure 7A: Sham+ebselen 7.07 ± 0.18 cm, *P*<0.0001 vs Sham+vehicle). Neither ebselen or vehicle treatment rescued increased faecal pellet numbers due to CS (Figure 1C: CS 4 ± 0.5, Figure 7B: CS+vehicle 5 ± 0.6, CS+ebselen 4 ± 0.5, *P*>0.05). In contrast, vehicle alone (i.e. CM-cellulose) increased the number of faecal pellets in sham-exposed mice, whereas ebselen administration with vehicle had no effect (Figure 1C: Sham 2 ± 0.4, Figure 7B: Sham+vehicle 5 ± 0.4, Sham+ebselen 4 ± 0.5, *P*<0.0001). Ebselen treatment did not affect small intestinal length (Figure 7C: Sham+ebselen 38.04 ± 0.65 cm vs CS+Ebselen 38.54 ± 0.69 cm, *P*=0.954).

## Discussion

In the present study, we found that CS exposure affected gut motility and resulted in morphological changes in the muscularis macrophages and neurochemical coding within the mouse GI tract. Gut dysmotility was improved by ebselen suggesting that targeting oxidative stress and inflammation could be a viable therapeutic strategy for GI dysfunction in patients with COPD.

It is well established that shortening of the colon and/or the small intestine occurs alongside intestinal inflammation [36–38]. CS exposure increased the number of faecal pellets within the dissected colon similar to previous reports in animal models of anticancer chemotherapy-induced gut dysfunction [32]. As previously reported, exposure to CS causes an initial decrease in body weight and subsequent growth retardation [26]. This body weight loss is believed to be a result of lower food consumption [23,39,40], but could also be because of altered digestion/absorption and/or GI motility. Our finding that changes in the colon length were inversely proportional to faecal pellet numbers needs further exploration. It is possible that the increased number of faecal pellets in CS-exposed animals reduced colon length due to the physical properties of the gastrointestinal tract. However, this relationship needs to be investigated in future studies by measuring the colon lengths after carefully removing the faecal pellets. Similar to another study investigating histopathology in C57BL/6 mice exposed to CS [9], we observed no significant changes in villus height or crypt depth in BALB/c mice following CS exposure. These authors also reported a significant inflammatory phenotype with Crohn’s disease-like inflammation observed in the colon and ileum in line with our observation of reduced colon length, a common indicator of an inflammatory state. The literature on exposure to CS and intestinal mucus levels is contradictory, with either increased or no change in mucus layer thickness reported following CS/nicotine-exposure [10,11]. Here, we report a reduction in colonic mucus layer thickness following 2 months of CS-exposure, which correlates with altered colonic motility. This aligns with reports suggesting alterations to the colonic mucus layer due to a mutation in the Muc2 gene lead to colonic dysmotility [41].

Another novel finding from the present study is that prolonged CS exposure alters NOS immunoreactive neurons, a specific subpopulation of neurons in the ENS. Altered immune responses in animal models of inflammatory bowel disease including ulcerative colitis and in response to chemotherapy have been shown to change the neurochemical phenotype of NOS-expressing neurons [42–45]. Enteric NOS-expressing neurons release the main enteric inhibitory neurotransmitter, NO which induces smooth muscle relaxation within the GI tract. A reduction in NO levels will therefore increase the frequency of gut contractions, as reported previously [46]. In the present study, however, there was no change in NOS neuronal numbers after 2 months CS exposure, suggesting that another mechanism is responsible for the increase in CMMC frequency in these mice. Our observation of reduced muscularis macrophage density and sphericity following 6 months CS exposure could also indicate functional responses to pathological and immune insult as has previously been proposed for macrophages and microglia [47–49].

We observed an increase in the frequency of colonic contractions and a reduction in colonic diameter in mice exposed to 2 months of CS. Nicotine has been shown to act on enteric neurons expressing nicotinic ACh receptors to contract gastrointestinal smooth muscle [50] and therefore could also contribute to this increase in colonic contraction frequency. The observed reduction in colonic diameter following 2 months of CS exposure may be due to reduced NO levels in myenteric neurons or via a nicotine-dependent mechanism. Changes in colonic smooth muscle constriction have previously been reported in mouse models of disease [51] and are a likely contributor to gut health. In addition to potential effects on inflammation, the nicotine content in cigarette smoke could contribute to the changes in gut function we observed. Exposure to cigarette smoke results in inhalation of nicotine levels similar to that of smokers themselves [52], and there is a broad literature describing the complex mechanisms of action of nicotine relevant to gastrointestinal disease [53]. For example, nicotine reduces smooth muscle tone and contractile activity in human distal large bowel [54] and animal studies have demonstrated that nicotine produces smooth muscle relaxation in the gastrointestinal tract [55]**.**

We have previously shown that the antioxidant ebselen reduces cigarette smoke extract-induced protein oxidation of murine alveolar macrophages and inhibits CS-induced increases in BALF macrophages, neutrophils, proteolytic burden, and macrophage and neutrophil chemotactic factor gene expression [21]. Given that oxidative stress is a key driver of CS-induced lung inflammation, targeting this pathway may be a novel means to alleviate inflammation in GI impairment in smokers and patients with COPD. In the present study, we show that ebselen prevents a chronic reduction in colonic diameter in mice exposed to CS. We observed that the carboxymethyl cellulose vehicle alone reduced the CS-induced increase in CMMC frequency, but not the changes to colonic diameter. Previous reports similarly suggest that carboxy methyl cellulose is beneficial in mouse models of GI dysfunction; however, the biological mechanisms responsible are unknown [56]. The reversal of reduced colonic diameter in the presence of ebselen may occur due to the antioxidant role of ebselen or alternatively, via an NO effect, since ebselen has been shown to reduce CS-induced vascular dysfunction via actions on smooth muscle in the mouse thoracic aorta (Brassington et al, unpublished observations). Nevertheless, this concept needs to be further investigated to resolve conflicting evidence suggesting that ebselen inhibits eNOS activity [57]. Although CMC reversed CS-induced colonic motility changes, it caused a subtle inflammatory response with respect to colon length shortening that aligns with current literature on C57BL6 mice where 1% CMC supplementation over 12 weeks leads to similar changes [58].

## Conclusion

In conclusion, we have shown that CS exposure causes gut dysmotility and altered muscularis macrophage morphology. We have shown for the first time that NOS-expressing enteric neurons alter their neurochemical coding in response to prolonged CS exposure, suggesting a direct effect of CS on enteric neurons and gastrointestinal function. Importantly, we have shown that the antioxidant ebselen rescues CS-induced changes in reduced colonic diameter in mice and may be a potential novel therapeutic for GI dysfunction in smokers or people with COPD.

## Clinical perspectives

- Cigarette smoking (CS) is the main cause of chronic obstructive pulmonary disease (COPD) and gastrointestinal (GI) dysfunction reduces quality of life for COPD patients. The aim of the present study was to investigate the effect of chronic CS on GI pathophysiology including gross anatomy and motility in mice. Potential beneficial effects of the antioxidant ebselen to reverse the GI phenotype were also investigated.
- The results of the present study showed GI anatomical changes indicative of GI inflammation. Cigarette smoking increased colonic motility and constricted the colon. Cessation of smoking for 10 days reversed the increase in motility but not the reduced colonic diameter. Moreover, ebselen treatment improved the CS-induced reduction in colonic diameter.
- This is the first study to show that chronic CS leads to GI dysmotility and that the antioxidant ebselen may be a potential therapeutic to treat CS-associated GI dysfunction.

## Supplementary Material

Supplementary Figures S1-S3

## Open Access

Open access for this article was enabled by the participation of University Of Melbourne in an all-inclusive *Read & Publish* pilot with Portland Press and the Biochemical Society under a transformative agreement with CAUL.

## Abbreviations

CMMC: colonic migrating motor complex
COPD: chronic obstructive pulmonary disease
CS: cigarette smoking
ENS: enteric nervous system
GI: gastrointestinal
NO: nitric oxide
NOS: nitric oxide synthase
TNBS: trinitrobenzenesulfonic acid
