# Supplementary material for: Ebselen prevents cigarette smoke-induced gastrointestinal dysfunction in mice
Source: Clin Sci (Lond). 2020 Nov 16;134(22):2943–57. doi: 10.1042/CS20200886 (PMC7676466; doi:10.1042/CS20200886)
Supplement: Supplementary Figures S1-S3 [file CS-2020-0886_supp.pdf]

Supplementary information

Supplementary figure 1

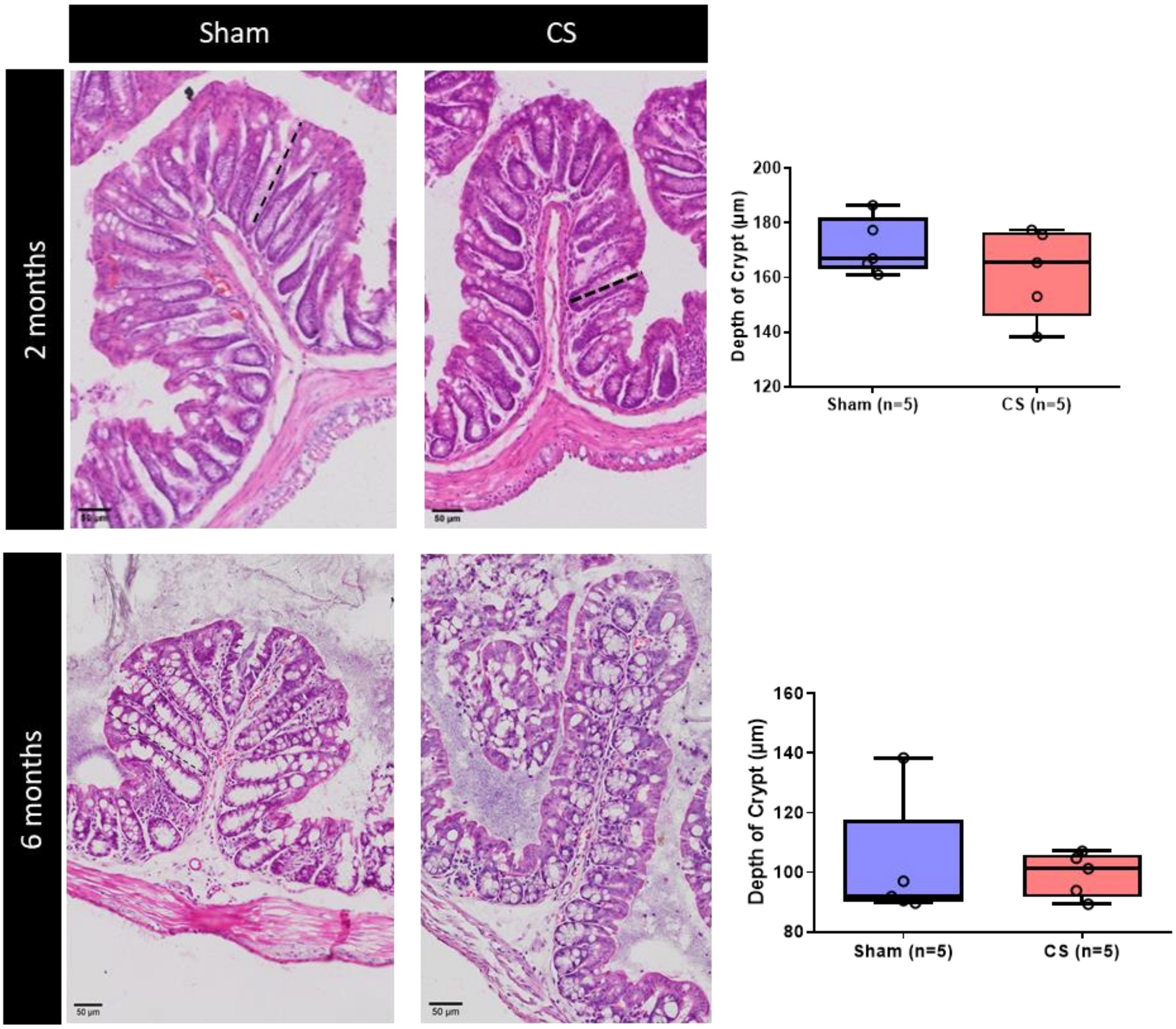

Typical histopathology was observed in proximal colon following both 2 months and 6 months of CS exposure

No significant changes were observed in crypt depth, villus height or histopathological scoring in proximal colon following 2-months and 6-months CS exposure (n=5, $p > 0.05$)

Supplementary Figure 2

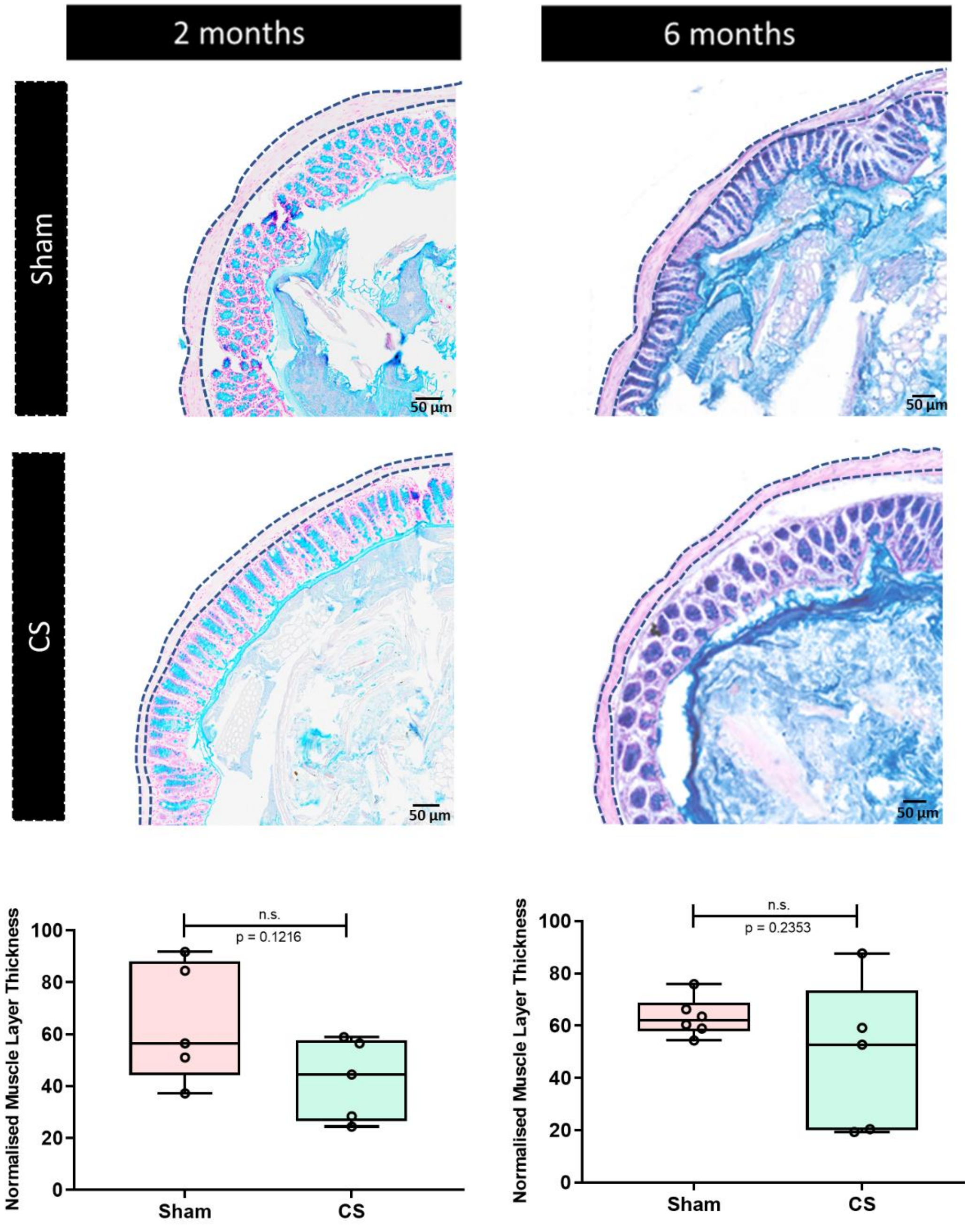

The area of the smooth muscle layer/unit length of the perimeter of the mid colon was not affected by both 2 months and 6 months of exposure to CS.

Supplementary Figure 3

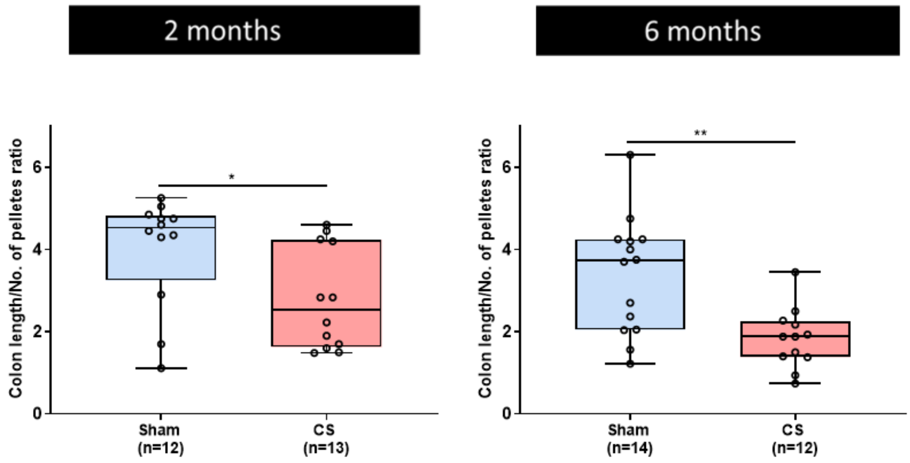

Both 2 months and 6 months CS exposure leads to reduced colon length to number of faecal pellets ratio. *p <0.05 and **p <0.001
